# Small Ruminant Lentiviruses (SRLVs) Break the Species Barrier to Acquire New Host Range

**DOI:** 10.3390/v5071867

**Published:** 2013-07-23

**Authors:** Juliano Cezar Minardi da Cruz, Dinesh Kumar Singh, Ali Lamara, Yahia Chebloune

**Affiliations:** 1 Laboratory Animal Health, Embrapa Goat and Sheep Research Center, CP 145, CEP 62010-970, Sobral, Ceará, Brazil; 2 Department of Life Sciences, Winston-Salem State University, Winston Salem, NC 27110, USA; 3 Veterinary Superior National School of Algiers, Route de Beaulieu-El Harrach-Alger 16200, Algeria; 4 Laboratory Pathogenesis and Lentivirus Vaccination, PAVAL Lab, Université Joseph Fourier Grenoble-1, Bat. NanoBio2, 570 rue de la Chimie, BP 53, 38041-Grenoble Cedex 9, France

**Keywords:** SRLV, cross-species, wild ruminants, adaptation, genetic diversity, recombination, primate lentivirus

## Abstract

Zoonotic events of simian immunodeficiency virus (SIV) from non-human primates to humans have generated the acquired immunodeficiency syndrome (AIDS), one of the most devastating infectious disease of the last century with more than 30 million people dead and about 40.3 million people currently infected worldwide. Human immunodeficiency virus (HIV-1 and HIV-2), the two major viruses that cause AIDS in humans are retroviruses of the lentivirus genus. The genus includes arthritis-encephalitis virus (CAEV) and Maedi-Visna virus (MVV), and a heterogeneous group of viruses known as small ruminant lentiviruses (SRLVs), affecting goat and sheep. Lentivirus genome integrates into the host DNA, causing persistent infection associated with a remarkable diversity during viral replication. Direct evidence of mixed infections with these two closely related SRLVs was found in both sheep and goats. The evidence of a genetic continuum with caprine and ovine field isolates demonstrates the absence of an efficient species barrier preventing cross-species transmission. In dual-infected animals, persistent infections with both CAEV and MVV have been described, and viral chimeras have been detected. This not only complicates animal trade between countries but favors the risk that highly pathogenic variants may emerge as has already been observed in the past in Iceland and, more recently, in outbreaks with virulent strains in Spain. SRLVs affecting wildlife have already been identified, demonstrating the existence of emergent viruses adapted to new hosts. Viruses adapted to wildlife ruminants may acquire novel biopathological properties which may endanger not only the new host species but also domestic ruminants and humans. SRLVs infecting sheep and goats follow a genomic evolution similar to that observed in HIV or in other lentiviruses. Lentivirus genetic diversity and host factors leading to the establishment of naturally occurring virulent *versus* avirulent infections, in addition to the emergence of new strains, challenge every aspect of SRLV control measures for providing efficient tools to prevent the transmission of diseases between wild ungulates and livestock.

## 1. Introduction

**Figure 1:**
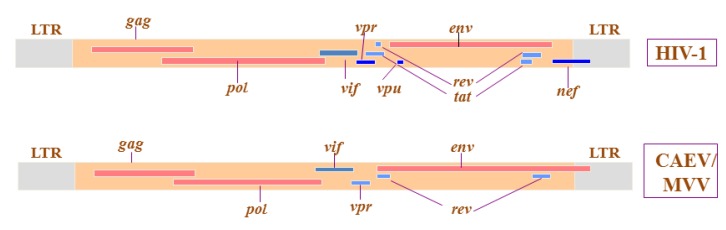
Organization of HIV-1 and CAEV/MVV lentivirus genomes. Both genomes have a 5' LTR and 3' LTR at each of their extremities and the structural and enzyme *gag*, *pol*, and *env* genes common to all retroviruses. In addition, HIV-1 genome carries six open reading frames (*vif*, *vpr*, *vpu*, *tat*, *rev* and *nef*) that encode regulatory and accessory proteins. Both CAEV and MVV genomes have only three additional open reading frames (*vif*, *vpr* and *rev*).

Lentiviruses are causative agents of many emerging and re-emerging infectious diseases threatening both animals and humans [1]. These positive single-strand RNA viruses are grouped in the subfamily *Lentivirinae* of the *Retroviridae* family. Retroviruses have been found in all classes of vertebrate animals, including fish, amphibians, birds and mammals [2]. In contrast, lentiviruses naturally infect a limited range of mammalian hosts including humans (HIV), non-human primates (SIV), felines (FIV), cattle (BIV), horses (EIAV), and small ruminants (SRLV): caprine arthritis-encephalitis virus (CAEV) and Maedi-Visna virus (MVV) [3]. The lentivirus genome (Figure 1) integrates into the host DNA and displays a remarkable genetic diversity promoted by high mutation and recombination rates during viral replication [4]. One of the archaic hallmarks of lentiviruses is their host specificity. Most lentiviruses infect only a limited number of host species. For example, HIV infects only chimpanzees and humans [5], or Equine Infectious Anemia Virus only horses. But recent evidences, particularly in the case of small ruminant lentiviruses (SRLV), suggest that such species barriers can be disregarded, as some of the emerging viruses are able to cross-infect closely related small ruminants.

Small ruminant lentiviruses (SRLVs), comprising arthritis-encephalitis virus (CAEV) and Maedi-Visna virus (MVV) are a genetic continuum of lentiviral species that were initially isolated from goats and sheep, respectively [6]. Following a prolonged incubation period they are responsible for a persistent infection which induces chronic degenerative disease of the joints, the lungs, the udder and central nervous system of infected hosts [7].

The evidence of a genetic continuum of caprine and ovine field isolates from various geographical areas, that did not simply cluster according to the animal species from which they were isolated, demonstrates the emergence of new viral recombinants capable of overcoming species barrier and resulting in cross-species transmission/infection. Direct evidence of mixed infections under field conditions with these two closely related SRLVs was found, as suggested by detection of some subtypes in both sheep and goats [8]. In naturally infected goats, persistent infections with both CAEV and MVV have been described, and viral chimeras generated by recombination between these variants have been detected [9].

Many emerging viruses partly escaping detection are already circulating in various hosts worldwide [10]. This is demonstrated by the identification of new SRLV subtypes, the description of cross-species infection and adaptation to wild animals, and recombinant viruses generated in double‑infected animals [9,11].

**Figure 2:**
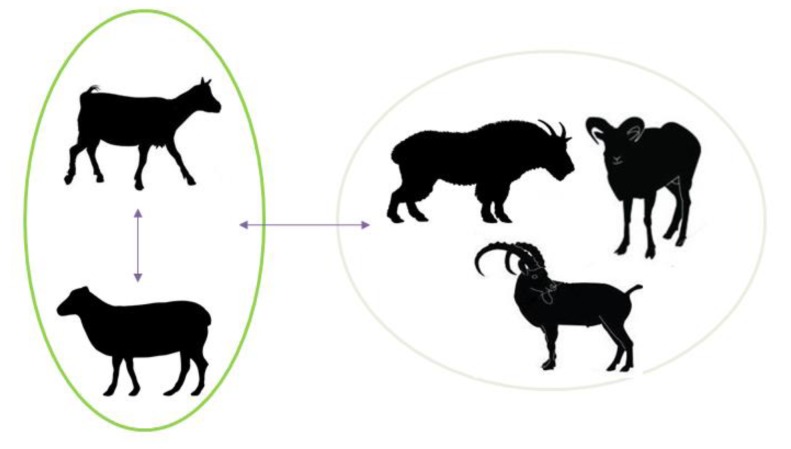
Cross-species transmission of SRLVs in domestic and wild ruminants. CAEV and MVV cause natural infection, adaptation and induced pathogenesis in both domestic sheep and goats (left). Experimental and natural cross-species infections were recently documented in wild small ruminants (right).

SRLVs affecting wildlife have already been identified, demonstrating the existence of emergent viruses adapted to new hosts [12]. These divergent viral forms capable of crossing species barrier to cause productive infections outside domestic small ruminants (Figure 2) are an invaluable model system to investigate questions of SRLV viral host-tropism, their evolution and transmission [13,14]. 

**Figure 3:**
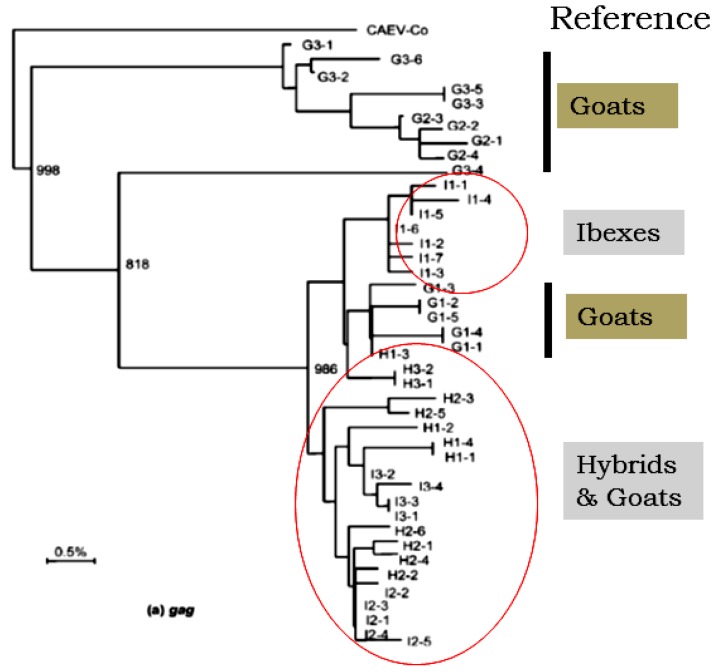
Example of phylogenetic analysis of CAEV sequences in goats and following spill over in goat/ibex hybrids and in wild ibex. Sequence analyses in *gag* gene from naturally infected goats (G), hybrids (H) and wild ibex (I) were used for alignment and phylogenetic analysis. The prototypic molecular clone CAEV-co was used as reference.

Although controversial, it is debated that SRLVs infecting sheep and goats follow a genomic evolution similar to that observed in HIV or in other lentiviruses [15].Viruses adapted to wildlife ruminants may acquire novel biopathological properties which may endanger not only the domestic ruminants but also new host species [11,14]. Lentivirus genetic diversity and host factors leading to establishment of naturally occurring virulent *versus* avirulent infections, and emergence of new strains, pose a serious challenge to every aspect of SRLV control measures used to prevent the transmission of diseases between wild ungulates and livestock. Phylogenetic analysis and characterization of the biological properties of SRLVs adapted to wildlife (Figure 3) and to new hosts may lead to development of advanced strategies in disease prevention and effective vaccine development.

## 2. Implications of SRLV Genetic Diversity and Cross-Species Transmission in Control and Eradication Strategies

The main factors in the worldwide spread of retroviruses are the rapid evolution and genetic variability associated with virus replication in the host. Other contributing elements are viral latency, viral persistence within infected host, inability of immune system to mount sustained immune responses against shifting targets due to antigenic drift and the failure of correct diagnosis due to antigen diversity. All these factors are the main drawbacks to the development of effective and safe vaccines. Although SRLVs share conserved genome organization and similar pathogenic mechanisms [16], there are clear genetic differences between many SRLVs strains [17,18,19]. The CAEV-Co and MVV-K1514 strains represent the prototypic viruses first isolated in goats and sheep, respectively [20,21]. Despite the knowledge of the high degree of genomic variation, little sequence information is available on virus variants circulating among different hosts worldwide [22]. The variability of SRLVs was first evidenced by the demonstration of antigenic variation in naturally or experimentally infected small ruminants [2]. 

SRLVs were reclassified into five principal sequence groups, A–E based on analysis of a genetic sequence comprising 1.8 Kb of the *gag-pol* region [8,22]. Group A is comprised of a large heterogeneous group that clusters around the prototype ovine lentivirus isolates: SA-OMVV (South African Ovine Maedi-Visna virus) [23], MVV K-1514 (Iceland) [21] and MVV EV1 (Scotland) [24]. Group A has been further subdivided into 10 distinct subtypes, A1–A10 [8,25,26]. Group B contains viruses related to the prototype goat lentivirus, CAEV Cork (USA) [27]. This group B has been further subdivided into three distinct subtypes, B1, B2 and B3 [8,28]. Virus isolates from goats in Norway, comprise group C [29], while those from Switzerland and Spain comprise Group D [8,30]. Finally, virus isolates from goats in Northern Italy and Sardinia comprise group E [23,31,32]. 

Like others retroviruses, SRLVs are positive RNA viruses that copy their genomes into complementary DNA during their replication [3]. The virion contains two copies of RNA, and the genes encoding virus proteins are organized in three major regions of the genome: the *gag* (*group specific antigen*), *pol* (*polymerase*) and *env* (*envelope*) genes. The SRLVs which encode auxiliary genes such as *vpr*, *rev* and *vif* are associated with the non-structural proteins with regulatory/accessory functions in viral replication [20]. Unlike primate lentivirus (HIV and SIV), SRLV also contains a non-coding sequence called LTR (long terminal repeat) located at each extremity of the genome (Figure 1), and SRLV genomes are under the control of LTRs with constitutive promoters that are independent from Tat transactivation [33]. The open reading frame that was initially named *tat* and shown to transactivate LTR promoters [34] was recently demonstrated to share more similarity and functions with *vpr* gene of HIV/SIV [31] and lacks the transactivation functions of SRLV LTR promoters. 

The extent of genetic diversity depends on a set of factors, but variation arises ultimately through two major mechanisms: Mutation and recombination. The *pol* gene encodes the enzymatic activities of reverse transcriptase (RT). RT is a RNA-dependent DNA polymerase that is essential for the conversion of viral RNA into DNA [2]. Most retrovirus mutations are introduced during the reverse transcription stage of the viral life cycle. During the replication of retrovirus genomes there is a high error rate as a result of the lack of proofreading activity of the RT [6].

Recombination is a complementary mechanism of genetic variation which can assemble the beneficial genetic combinations more efficiently than mutation alone. Insertions and deletions in the lentivirus genome are also frequent occurrences due to high rates of virus replication [35]. Together, these mechanisms create a rapid generation of genetically diverse viral populations which in turn are subjected to selection pressures by the host restriction factors and immune response [36]. 

On the other hand, dual infections (coinfections or superinfections) are an essential status for such recombination events [4,9]. Cross-species infections may occur in animals living under natural conditions, which suggest that coinfections with SRLVs are possible [8,9,13]. A previous study has provided clear evidence of A/B strain recombination within the *env* gene in an infected goat [9].When a host cell becomes coinfected by two or more different SRLVs, their genomes may become co‑packaged into viral progeny. A recombination event may take place as a result of the co-packaging of two copies of RNA genome into each replicated virion [4]. The influence of this genetic evolution on the biological properties of SRLVs may lead to important adaptive changes in the context of SRLV pathogenesis and virus transmission [7]. Although recombination is one of the characteristics of the HIV infection, more studies are needed for SRLVs. Many questions, that make assessment of the role of viral diversity and its consequences on control and eradication strategies very difficult, still remain to be answered.

In several countries, SRLVs strains have been analyzed and compared with current prototype strains of CAEV and MVV [17,18,22,37,38,39]. Although it is generally appreciated that viral diversity enables lentiviruses to evade the host immune system [4,5], the severity of SRLVs clinical implications remains unknown. In domestic small ruminants, approximately 30% of the infected animals develop clinical signs, mainly due to chronic arthritis and progressive interstitial pneumonia and mastitis [7]. However, in the early 1930s a devastating outbreak of MVV in Iceland resulted in the death of over 100,000 sheep. It also led to the slaughter of more than half a million animals in order to eradicate MVV [16].

But outbreaks of disease with virulent strains kept re-emerging in susceptible populations in various parts of the globe. More recently, outbreaks with virulent strains associated with extensive neurological diseases were found in sheep of the central area of Spain [40,41,42]. In this area there are more than four million sheep and about 170,000 goats in which numerous cases of neurological form of diseases have been diagnosed. Affected animals present histological lesions that resemble those previously described in Icelandic sheep. The spinal cord of affected animals appears to be the main target of lesions [40,41,42]. Furthermore, the disease affects young and adult animals, with high mortality rates causing significant production losses [40]. Sequence data of isolated virus genomes showed that they belong to genotype A2/A3, and *in vitro* cultivation properties revealed high virus replication efficiency in blood monocyte-derived macrophages but a slow/low virus replication in fibroblast-like cells. Virus replication/production could not be the explanation of their increased virulence [10]. The inherent different biological characteristics between subtypes may be correlated with clinically asymptomatic disease, increasing the potential for unwitting transmission [42,43,44,45]. The long period of SRLV latency and incubation before development of clinical signs also results in wide virus dissemination where asymptomatic but infected individuals act as insidious transmitters and carry SRLVs to different geographical locations [45]. 

Most transmissions of SRLVs among sheep or goats occur through the ingestion of virus infected colostrum [28,44,45,46]. In the US, many strains of ovine lentiviruses (OLVs) that have been isolated from naturally infected sheep were found to be genetically closer to the goat lentivirus CAEV rather than the sheep lentivirus MVV [47,48]. This may result from cross-species infection and adaptation of CAEV from goats to sheep, since in the US sheep are raised mainly for meat and orphan lambs and those born from ewes not producing enough milk are often fed with colostrum and milk from goats. However, these virus isolates retained their macrophage tropism and can be adapted to non-macrophage cells *in vitro* following co-cultivation of infected macrophages with the non-permissive target cells [49]. In goats, a recent sequence analysis study of DNA and RNA, comprising the V4–V5 region of *env* gene, showed a different distribution of SRLV variants between blood and colostrum [37]. Different body compartments may subject a virus population to evolutionary selection pressures imposed by host restriction factors and immune response. High recombination and mutation rates subject to an immune selection pressure during viral replication may create a viral diversity between body compartments which in turn may impact, in this case, the efficiency of lactogenic transmission.

Genetic diversity also poses a relevant problem for SRLVs, testing and diagnosis [44,49]. The absence of SRLV diagnotic protocols that are able to detect all groups and subtypes of the virus, results in failure to detect many infections [44,50,51]. Performance of the currently available serology and molecular diagnostic tests is dependent upon a number of factors, including the percentage of identity between the nucleotide sequences of viral populations circulating in infected flocks of a certain geographic region and the sequences used to generate SRLV tests [44,49]. 

SRLVs have been detected by ELISA, PCR and *in situ* hybridization in semen and various tissues of male genital tract suggesting a potential source of transmission [52,53,54,55]. Semen contaminated with viral agents may change the epidemiology of lentivirus diseases since it could infect numerous farms, areas, or even countries in a short period of time [56,57]. Molecular epidemiology revealed that Swiss B1 strains differed no more from Brazilian, French or US strains than from each other, suggesting virus propagation through international livestock trade [18,22].The present situation not only complicates animal trade between countries but also favors the risk that highly pathogenic variants may emerge as already observed in the past.

SRLV’s mutagenic potential and diverse assortment of antigen epitopes have hampered vaccine development efforts [58]. Immunizations with viral clones, genetically modified viruses or recombinant plasmids have become more recent alternatives to classical immunization [59,60,61]. Nonetheless, the role of antibodies in SRLV immunity is complex [61]. SRLVs induce relatively few neutralizing antibodies, and finding ones with broad reactivity has been difficult [58]. Despite the evidence of providing some protection among vaccinated animals, the immunization strategies used so far have shown a limited efficiency hampering their broader application. Due to these limitations, current vaccination strategies are not suited for successful application under field conditions. [58,59,60,61,62].

SRLVs are widespread throughout the world causing serious economic implications for farmers and animal industry. SRLV infected animals produce less milk of poor quality, lose weight and are often prematurely culled [63]. The resulting economic losses related to the widespread distribution of SRLVs have led to the establishment of control and eradication programs for both sheep and goats in several countries [58,64,65].

There are millions of sheep and goats infected with SRLVs worldwide. The prevalence of SRLVs infections in North America and Europe ranges from 30%–80% compared to 0–10% in Africa and South America [38]. Consequently, SRLVs are classified as a major infectious disease of small ruminants by the (Organisation Internationale des epizooties) OIE. In spite of OIE’s efforts and many eradication programs operating in various countries, SRLV still remains a challenge for farmers and health authorities due to the absence of an efficient vaccine or a diagnostic tool capable of detecting all SRLV subtypes [50,66].

Besides the high prevalence of infection in many countries, another major obstacle to eradication of SRLV is the ability of SRLVs to jump the species barrier. The first direct evidence came from natural interspecies transmission of SRLV subtype A4 between different animal species living under natural conditions [8]. The fact that SRLV subtypes have been isolated from both sheep and goats proved the hypothesis that cross-species transmission of SRLV can occur between close species living together, although the frequency and direction of such a cross-species jump remain to be elucidated [6,15]. As mentioned earlier, there is evidence of recombination between different SRLVs strains from groups A and B in dual infected goats [9].

Recombination of different strains and subtypes contributes significantly to viral diversity and evolution both within individuals and populations [4,27,67]. SRLV evolution and variability might facilitate cross-species infections and domestic goats and sheep carrying SRLVs may transmit it to other animal species [11,13]. The emergence of infectious diseases within a new host species or naïve populations can greatly influence species survival and adaptation [5,61,68]. Since sheep and goats may serve as reservoirs for all SRLV subtypes, a successful control program, therefore, requires inclusion of both species [23].

## 3. Aspects of SRLVs Emergence in Wildlife Species

Numerous emerging infectious diseases, including zoonoses, were shown to originate from wildlife [69]. Degradation and fragmentation of wild spaces are the main anthropogenic factors associated with the emergence of diseases in wildlife [70]. As a consequence, the importance of interface between wildlife, livestock and humans is becoming a global issue of growing interest. The best example of this phenomenon is the emergence of HIV/AIDS in humans that has provided the proof of the dramatic consequences of cross-species infections on public health [71]. Strong evidences indicate that HIV emerged in humans following multiple zoonotic transmissions of simian immunodeficiency virus (SIV) from non-human primates (NHPs) that probably occurred in the process of hunting and butchering of primates for bush meat. The capture, trade and keeping of monkeys as pets [35,68,72] also facilitated contact between SIV carrier monkeys and immunological naïve human hosts on a regular basis leading to cross-species adaptation of SIV that later developed into HIV.

Independent zoonotic transmission events from NHPs to humans have generated two major virus types HIV-1 and HIV-2. HIV-1 remains a major global health problem with an estimated 33.3 million people living with the disease and over 30 million that have since died [5] due to AIDS [5]. This has provided the clear demonstration that lentiviruses have high potential for crossing the species barrier, and adaptation to new host species with increased virulence. Similarly, natural infection of goats with MVV and sheep with CAEV has been reported, suggesting the lack of an efficient species barrier that could prevent cross-species infections [29,64].

Small ruminant lentiviruses (SRLVs) have a number of features in common with HIV-1 such as genome organization, long preclinical period following transmission of the virus, latency and persistence with a slow development of chronic degenerative disease, which may gradually lead to death [16]. However, SRLV origins remain obscure [3], although some evidences indicate that CAEV initially isolated in goats probably emerged after cross-species transfer of a closely related sheep virus MVV in recent times [15]. 

Prevalence of SRLVs often reaches 100% among infected flocks [9,73,74,75,76]. Without the availability of robust diagnosis tools and an efficient vaccine eradication of disease and virus often fails [68,69,70]. Therefore, characterization of biological and pathological properties of SLRVs along with effective control measures and an effective vaccine are urgently needed [19,58].

Sheep, goats and cattle are major sources of high quality protein worldwide, and due to (I) rising incomes, (II) population growth, and (III) increased urbanization, there is a growing demand for meat and other animal products [77]. Because of increasing demands for safer and better food products by consumers, authorities and traders, farmers whose animals and herds have high prevalence of infections stand the risk of losing income and business due to infected animals. Such farms are in danger of being excluded from the animal product trade [78,79]. 

In most countries crude milk is used as the raw substrate for dairy products [80]. Since consumption of milk from infected animals is the main route of SRLV transmission [46], such practices may lead to distribution of SLRVs in hosts consuming contaminated milk. In HIV-1 infected patients an immunologic cross-reactivity between CAEV and HIV-1 antigens has been reported. It was suggested that this antigenic cross-reactivity occurred as a result of consumption of contaminated goat dairy products [81]. In a similar study, analysis of serum samples from children who consumed goat milk products found serological reactivity against CAEV gp 135 [82]. It is now well established that HIV/AIDS arose from human contact with SIV-infected NHPs [33]. In the case of CAEV, recent reports suggested that the absence of a functional CAEV receptor on human cells is the only barrier protecting human cells from CAEV infection *in vitro* [83]. In an elegant study, Chebloune and his colleagues demonstrated that when the CAEV genome was introduced inside the cell, or when human cells were infected with VSV-G-pseudotyped CAEV thereby bypassing the need of receptor-mediated endocytosis of the virus, CAEV was capable of replication in human cells that resulted in virus particle production and release of high titers of replication-competent pathogenic virus from transfected cells [83].

There is growing evidence of increased cross-species infection in mixed flocks worldwide resulting in the emergence of new variants adapted to new hosts and viral chimeras [69]. Domestic sheep and goats in many countries are heavily infected with these lentiviruses and may transmit these infections to other animal species [84]. In such systems, domestic animals were genetically selected for a specific production, and as a result, they are less adapted and resistant to a high exposure rate to pathogens [85].

Although incipient, there is still a possibility of persistence of SRLVs infection in human cells [86] that are routinely exposed to SRLV via ingestion of raw contaminated goat milk or milk products. Even though non-adapted host successfully inhibits normal viral replication [87], the frequent exposure to SRLV by routine ingestion of contaminated milk and dairy product may result in adaptation of SRLVs among humans in due course of time, on similar lines as SIV/HIV did [79].

Experimental infection of bovine calves with CAEV demonstrated that CAEV causes productive but not persistent infections in calves [14,86]. The CAEV infection was eventually cleared by calves but not before CAEV successfully replicated in bovine cells. These experiments suggest that the repeated incidences of host-virus interactions may result in emergence of a new pathogenic strain that will be able to cause disease among newly adapted hosts. A look at the recently emerged pathogens, mainly viruses, in wildlife [67] indicates possibility of such evolution and adaptation. Viruses, due to their high recombination and mutation rates during viral replication, are most likely to evolve and adapt to a new naïve host. RNA viruses from this category are perfect candidates for emergence in new hosts or naïve animal populations [1] during repeated virus-host interactions. Among RNA viruses, lentiviruses will be leading such evolution and emergence of new strains due to the lack of proof reading ability during reverse transcription and replication. In Europe, bluetongue virus (BTV) recently emerged and became enzootic in livestock [65,88]. Wild ungulates were proved to be receptive to the SRLVs in many regions and various diseases affecting new hosts have already been identified in wildlife [11,89]. The successful experimental and natural infections of wild small ruminants, such as ibex (*Capra ibex*) and mouflon (*Ovis gmelinii*) with SRLVs provide proof that these wild ruminants are susceptible for CAEV infection. Recovery of CAEV genetic sequences from Ibex that were in close proximity of grazing goat (Figure 3) lends further support to the hypothesis of cross-species adaptation of SLRVs [12,13]. Therefore, wildlife and livestock may represent major reservoirs of SRLV viruses from which cross-species infections may occur [90].

Recent detection of cross-species infection and adaptation of domestic ruminant lentiviruses in free wild ruminants have further cemented this argument [1,91]. In Rocky Mountain goats (*Oreamnos americanus*) a fatal naturally occurring CAEV infection was observed in four individuals. The CAEV was possibly transmitted to this species through ingestion of milk from infected domestic goats [11].

Despite the proven efficiency of lactogenic transmission, the pastures are places where the transmission rate of infectious diseases between domestic and wildlife ruminants is the highest [77]. The contact between infected domestic sheep and goats and wildlife ruminants is favored by practicing free grazing in the areas of rehabilitation of many wildlife ruminants following introduction of a number of animals from different species [90]. This practice promotes a close contact between different species and may result in cross-species transmission of these pathogens. In France, SRLV was detected in three ibexes that shared pastures in French Alps with a domestic goat flock [12].

Different factors can promote the constantly increasing interactions between wildlife and domestic animals. In Brazil, various endangered native goat breeds were infected with SRLVs through the introduction of exotic breeds to improve efficiency of milk production in local dairy herds [92,93]. Of note, endangered species or breeds are particularly vulnerable to reduction in effective population size due to disease [94].

In many countries, large populations of wild ungulates are concentrated in small delimited areas because of high human density and distribution [95]. In addition, these interactions are also favored by the growing numbers of farms raising wild ruminants together with domestic animals [96]. Hunting behavior of humans may indirectly increase the contacts between wildlife and livestock populations [77]. The uncontrolled hunting of predators led to their extinction and a subsequent imbalance of interactions between species. As a result of natural tourism, the frequency of contacts between humans and wild species is increasing [97]. Additionally, intrusions of scientists or veterinarians for monitoring wild populations, even if carefully controlled, may be a risk of disease transmission. Altogether, these interactions may favor the cross-species transmission and may result in the emergence of variants with unknown biopathological properties for animals and humans [72]. 

In order to successfully adapt to a new host, lentiviruses must evolve quickly to evade host defenses before they are transmitted to other individuals of the same species [87]. However, relevant clinical data from wild populations has not been collected in most cases due to the difficulty of sample collection and requirement of prolonged surveillance [11]. Therefore, the quantum of data about lentiviruses in wildlife ruminants is very limited [13]. Currently available diagnostic tests also fail to detect closely related wildlife viruses [12].

In domestic SRLV-free flocks, virus may re-emerge spontaneously. Serology tests in many cases also fail to detect antigenic variants by routine methods [98]. Likewise, diagnostic tools to detect these pathogens in wildlife ruminants are nonexistent, which may jeopardize the control of these viruses. In this context, novel diagnostic tools need to be developed in order to investigate lentiviruses in wildlife ruminants. Progress in understanding host-lentiviral interaction/adaptation, cross-species transmission and the biological properties of these emergent lentiviruses may facilitate development of better diagnostics and maybe even of vaccines to contain and control this emerging problem.

## 4. Conclusions

Wildlife and domestic animals are the major reservoirs for emerging and re-emerging pathogens, threatening both animals and humans [69]. Degradation and fragmentation of wild spaces by the anthropogenic action enhanced the contact frequency between different species and may facilitate the emergence of diseases in wildlife ruminants that may serve as uncontrolled reservoir of pathogens. Genetic diversity presented by lentiviruses, associated with cross-species transmission between sheep and goats together with the spill-over in wildlife ruminants, underlies the adaptive changes which may have consequences in the context of SRLV pathogenesis, immune response and diagnosis, virus transmission and vaccine development. 

Furthermore, the emergence of infectious diseases within new hosts species or naïve populations can greatly influence species survival and adaptation. In fact, the cross-species infection of simian lentiviruses from non-human primates to humans has resulted in one of the most devastating infectious diseases (HIV/AIDS) of the last century. This demonstrates how a new viral pathogen can pose serious threats to public health [33]. Recent data about SRLVs suggest emergence of recombinant and new variants, some of which escape detection with current diagnostic tools and are circulating in domesticated and wild ruminants. The potential risks of these emerging/recombinant viruses or increase in their pathogenicity or adaptation to a new host species are currently hard to predict. 

Cross-species transmission between domestic small ruminants and wild species may affect the pathogenic and tropism properties of SRLVs, jeopardizing health and environmental safety. A continuous surveillance of wild small ruminant populations may improve our knowledge of the relationship between genetic evolution and new biological properties of emergent viruses, which in turn could generate biomedical advances and improved strategies for lentiviruses control in humans, domestic and wildlife small ruminants.

Finally, the recent discovery that goats in Europe are readily infected with highly attenuated strains of CAEV that escape the detection tools and are not associated with disease development provides unique opportunity to use some of these strains as live-attenuated vaccines for goats to induce protection against the highly pathogenic strains. Since CAEV is not an infectious pathogenic virus in human, these highly naturally attenuated strains of CAEV could be used as a basis to derive live‑attenuated chimeric genomes as vaccines against the deadly HIV-1 in human.
